# Integrated Computational Approaches and Tools for Allosteric Drug Discovery

**DOI:** 10.3390/ijms21030847

**Published:** 2020-01-28

**Authors:** Olivier Sheik Amamuddy, Wayde Veldman, Colleen Manyumwa, Afrah Khairallah, Steve Agajanian, Odeyemi Oluyemi, Gennady M. Verkhivker, Özlem Tastan Bishop

**Affiliations:** 1 Research Unit in Bioinformatics (RUBi), Department of Biochemistry and Microbiology, Rhodes University, Grahamstown 6140, South Africa; oliserand@gmail.com (O.S.A.); wamiveld@gmail.com (W.V.); colleen.manyumwa06@gmail.com (C.M.); afrahkhairalla@gmail.com (A.K.); 2 Graduate Program in Computational and Data Sciences, Keck Center for Science and Engineering, Schmid College of Science and Technology, Chapman University, One University Drive, Orange, CA 92866, USA; agaja102@mail.chapman.edu (S.A.); odeye100@mail.chapman.edu (O.O.); 3 Department of Biomedical and Pharmaceutical Sciences, Chapman University School of Pharmacy, Irvine, CA 92618, USA

**Keywords:** Allostery, allosteric modulators, network analysis, MD-TASK, drug resistance, precision medicine

## Abstract

Understanding molecular mechanisms underlying the complexity of allosteric regulation in proteins has attracted considerable attention in drug discovery due to the benefits and versatility of allosteric modulators in providing desirable selectivity against protein targets while minimizing toxicity and other side effects. The proliferation of novel computational approaches for predicting ligand–protein interactions and binding using dynamic and network-centric perspectives has led to new insights into allosteric mechanisms and facilitated computer-based discovery of allosteric drugs. Although no absolute method of experimental and *in silico* allosteric drug/site discovery exists, current methods are still being improved. As such, the critical analysis and integration of established approaches into robust, reproducible, and customizable computational pipelines with experimental feedback could make allosteric drug discovery more efficient and reliable. In this article, we review computational approaches for allosteric drug discovery and discuss how these tools can be utilized to develop consensus workflows for *in silico* identification of allosteric sites and modulators with some applications to pathogen resistance and precision medicine. The emerging realization that allosteric modulators can exploit distinct regulatory mechanisms and can provide access to targeted modulation of protein activities could open opportunities for probing biological processes and *in silico* design of drug combinations with improved therapeutic indices and a broad range of activities.

## 1. Introduction

Allosteric regulation is often a mechanism of choice for proteins and biomolecular assemblies to operate in complex signalling cascades and to modulate their activity levels, adapting to binding partners in the cellular environment during signal transduction, catalysis, and gene regulation [1,2,3,4,5]. The advances in X-ray crystallography, Nuclear Magnetic Resonance (NMR), and biophysical techniques have enabled numerous detailed investigations of large protein systems and conformational dynamic processes at atomic resolution [6,7,8,9,10,11,12,13,14,15,16,17,18,19]. These developments have facilitated the integration of computational and experimental studies of allosteric regulation, eventually leading to new conceptual outlooks and attempts to develop a unified theory of this allosteric phenomenon. The thermodynamics-based conformational selection model of allosteric regulation has been particularly fruitful in explaining a wide range of experiments by assuming that a statistical ensemble of preexisting conformational states and communication pathways is inherent to any protein system and can be modulated through allosteric ligand perturbations [20,21,22,23,24,25,26]. While great leaps have been made in the field of molecular modelling, NMR spectroscopy, and X-ray crystallography, it should be noted that no single method can provide allostery information for all cases due to the complexity and incomplete understanding of allosteric phenomena.

Understanding molecular mechanisms of allosteric regulation in proteins has attracted considerable attention in both academia and industry owing to the importance of discovering allosteric modulators of therapeutically important targets [27]. These efforts are motivated by fundamental differences in structural and evolutionary diversity between active and allosteric sites even among structurally similar proteins of the same family. While active sites for structurally related proteins and protein families are often highly conserved and present a formidable challenge for design of selective modulators, allosteric binding is typically more dynamic and structurally and evolutionarily diverse, thereby often alleviating conceptual difficulties in the design of target-specific therapies and addressing lingering problems of toxicity and side effects [28]. Another important incentive for the development of allosteric drugs is that, while traditional orthosteric drugs usually inhibit protein activity, allosteric modulators may not only inhibit but also increase protein activity (allosteric activators) [29]. In the last decade, drug discovery has been shifting its focus toward targeting allosteric sites in order to improve compound selectivity [28,29,30,31,32,33]. Allosteric drugs also feature distinct physicochemical properties, adding further freedom for discovery of novel active compounds, and can often be combined with orthosteric drugs into synergistic drug cocktails to modulate and improve enzyme activities, specificity, and pharmacological profiles.

While orthostery-based therapies have enhanced the quality of life for patients, they have brought forth many daunting challenges for which allostery may provide new solutions. Drug discovery against more diverse protein targets can result in less toxic and more specific therapies. The incorporation of dynamic and network analysis tools has proven their effectiveness in drug discovery studies of several target proteins [32,33,34,35] and offer a promising direction for the analysis of large datasets [36]. With the maturation of open-source projects, the availability of cheaper computation, and large datasets, in silico simulations are a very attractive venture for early-stage drug discovery as they offer cost-effective drug development. The integration of such approaches into robust, reproducible, and customizable workflows should make in silico allosteric drug discovery more efficient and reliable. In this review article, we discuss how the integration of state-of-the-art structural, dynamic, and network-based approaches for simulation of ligand–protein binding can provide a comprehensive methodological framework for advancing computer-aided discovery of allosteric sites and allosteric modulators of protein functions and mechanisms.

## 2. Part I: Overview of Allostery and Allosteric Drugs

### 2.1. What Is Allostery?

Allostery is generally defined as a reversible functional and conformational modulation at one site resulting from a remote perturbation in a protein [27,37,38]. These remote events can be instantiated from both covalent (residue mutations and chemical reactions) and non-covalent (intermolecular interactions) events and are well-summarized by Nussinov and Tsai [27]. The phenomenon is also extended to include entropic changes, which may prevail even when no conformational change may be apparent [39,40,41]. Allostery-driven conformational changes are not expressed as two discrete conformational transitions but rather exist as an equilibrium comprising a population of various conformations [42]. More generally, allostery is an inherent property of biomacromolecules [43] and its dysfunction is linked to the cause of several diseases [27]. Two examples of inherent allosteric mechanisms include the protein binding by a hormone to drive conformational changes that affect protein–protein interactions in signalling cascades and small molecules regulating catalytic activity by binding at loci far from an active site [44].

### 2.2. Understanding Allosteric Mechanisms Using Existing Approaches

Allosteric effects are not easily detectable by any single method as they can take many forms, and a diverse pool of conformational samples is often needed to expose these rare events [41,45,46,47]. Current understanding of the details of allosteric mechanisms is still fragmentary [48]. However, the recent experimental breakthroughs in NMR technologies have enabled structural studies of large protein systems and conformational dynamic processes at atomic resolution, providing unique insights into allosteric mechanisms [12,14,15,17,18]. While studies of allosteric regulation often emphasize thermodynamic aspects of the mechanism and incorporate a population-shift conformational selection paradigm, the critical role of conformational dynamics delineated in NMR studies led to the development of the “dynamics-driven” framework of allosteric phenomenon. In dynamics-driven allostery, effector ligands can induce allosteric effects through global redistribution of protein fluctuations and can propagate signals through dynamic modulation of functional motions even in the absence of visible structural changes [5,13,49,50,51,52]. Recent time-resolved infrared spectroscopy experiments have indicated that allosteric transitions occur on multiple timescales. A time-dependent view of allosteric communication revealed that allostery can be manifested by hierarchical propagation of structural and dynamical changes, suggesting a high degree of conformational heterogeneity of the ensemble of communication routes in proteins [53,54]. Relaxation dispersion NMR methods have also enabled the detection of rapid conformational exchanges between ground and excited states occurring on the μs–ms timescale that facilitated characterization of hidden excited states that play a significant role in dynamic modulation of protein function and allosteric mechanisms [14,15]. Structural identification and characterization of lowly populated states by high-pressure NMR can allow for detection of reversible transitions under thermodynamic equilibrium conditions that are functionally relevant for allosteric mechanisms [55,56]. Pressure-dependent chemical shifts may also measure redistributions in conformational entropy and specify dynamic allosteric mechanisms, offering an exciting experimental platform for design of allosteric modulators specifically targeting lowly populated functional states [55,56,57].

Allosteric interactions and communications can be conveniently described and characterized by dynamic networks of interactions between components of biological systems. The organization and evolution of dynamic residue interaction networks in proteins allows for formation of ensembles of pathways that transmit signals by propagating conformational fluctuations and functional motions between distant sites. Recent years have witnessed the development of various approaches that investigate NMR chemical-shift perturbations to identify allosteric networks in proteins [58,59,60,61,62,63].

The ensembles of allosteric communications and protein residues involved in signal transmission via population-shift or dynamics-based allostery can be experimentally examined by NMR spectroscopy [64,65,66]. NMR chemical shift responses to bound ligands are commonly employed as diagnostic tools for identifying coupled networks within allosteric proteins that could quantify potential communication pathways [14,50,67].

NMR chemical exchange saturation transfer (CEST) experiments [68,69,70] can identify invisible hidden states and characterize slow-to-intermediate conformational exchanges. NMR chemical-shift covariance (CHESCA) and projection (CHESPA) analyzes can identify residue interaction networks that show correlated changes in chemical shifts due to allosteric perturbations caused by ligand binding or mutations [71,72,73]. NMR chemical-shift perturbations have also been combined with Markov modelling and network analysis to reveal the dynamic flow of communication between allosteric communities in proteins [74].

There is increasing evidence that dynamic allostery may be a common feature in many protein interactions, and as a result, allosteric mechanisms are no longer viewed as being mediated solely through structural transitions that select specific stable conformational states.

Even though NMR technologies opened the doors to us to greatly understand the mechanisms of allostery, over 80% of the structures available from the Protein Data Bank (PDB) comprise macromolecules solved by X-ray crystallography [75]. These are a representation of the most frequent conformations adopted by proteins from their respective pools of conformation ensembles [76]. As allosteric sites are rarely observed in crystallographic structures, a large amount of conformational sampling may be needed to uncover such high-energy states. For instance, cryptic (or hidden) allosteric sites sporadically appear during conformational transitions of a protein in the presence of a bound ligand. They are a recently discovered form of allostery whereby ligands can bind to pockets that are not present in crystal structures [77]. These “hidden” allosteric sites are essentially invisible in crystal structures, apart from some chanceful detection due to the stabilization of the rarer, higher-energy conformation by certain compounds [78]. Fortunately, an abundance of computational tools have been designed over the years to examine allosteric mechanisms in protein systems [36]. In silico conformation sampling techniques allow us to unveil such rarely observed protein conformations [78] by simulating protein motion, thus facilitating the discovery of potentially druggable sites [79]. Exhaustive sampling is impossible, and highly precise quantum mechanics (QM) simulations are limited to small systems or portions of larger systems. Coarse-grained molecular dynamics (MD), markov state models (MSMs), and elastic network models (ENM) offer speed improvements by reducing complexity of all-atom protein representation while maintaining topological and network integrity of protein systems that drive allosteric mechanisms and global regulatory functions. The explored approaches have included normal mode analysis (NMA) [80,81,82,83], MD [84], and machine learning [85]. Brown and coworkers successfully coupled full-atom MD simulations to dynamic residue network (DRN) analysis to study allosteric effects in disease-causing variants [86]. These computational approaches will be further discussed in the second part of this review article.

### 2.3. Understanding the Allosteric Effects of Disease and Drug-Resistant/Sensitive Mutations—Precision Medicine

Human diseases and traits have been associated with single nucleotide polymorphisms (SNPs) through the use of genome-wide association studies (GWAS) [87,88]. There is even considerable genetic variation between just two random individuals, where 10,000 non-synonymous single nucleotide polymorphisms (nsSNPs) were found to exist between their exomes [89]. These mutations have the potential to alter distal functional sites of enzymes by means of allosteric signalling—where the dynamics of the entire structure is transformed as a result of the mutation [90,91,92]. Fortunately, progress in allosteric research has made it possible to determine allosteric molecular mechanisms in a myriad of allosteric systems in much detail [93]. For example, network analysis is usually performed to determine the pathways that connect the mutation and the active site and aids in the study of allosteric communication [86,94,95]. A recent publication studied six validated non-synonymous single nucleotide variations (nsSNVs) to identify underlying mechanisms responsible for CA-II deficiencies resulting in the phenotype of osteopetrosis with renal tubular acidosis and cerebral calcification [96]. In this study, Sanyanga et al. combined MD and DRN [97] analysis and showed that nsSNVs have indirect/allosteric effects, providing greater insights into SNV mechanism of action. Hence, the study proposed taking steps towards the treatment of CA-II deficiencies. Further, the authors highlighted the importance of studying missense mutation effects in proteins with combined approaches and, hence, in a broader sense, precision medicine-related research. This computational approach is detailed in SubSection 3.3.

Precision medicine combines pharmacology and genomics to exploit genetic variation in the human population in order to deliver innocuous but powerful drugs to certain groups of individuals. This is based on the idea that an individual’s genetic makeup determines their reaction to a drug, good or bad [88,98]. For instance, mutations can be linked to altered drug sensitivities in patients [99,100,101]. This opens the door to personalized medicine, where knowledge of drug-resistant and drug-sensitive SNPs can assist in the development of effective biomarkers [99] and allow treatments to be tailored to individual patients [102,103]. Furthermore, understanding structural changes caused by nsSNPs would enable the design of novel drugs to target these mutations and, thus, is key in advancing precision/personalized medicine [104,105]. One example can be given from stroke and stroke-related medications. Clopidogrel and Warfarin, antiplatelet/anticoagulant drugs, are widely used for primary and secondary prevention of stroke. It has been shown that genetic polymorphisms resulting in reduced function of cytochrome P450 2C19 (CYP2C19) were associated with increased cardiovascular risk and mortality in coronary artery disease patients on clopidogrel treatment. A study of clopidogrel response in the Amish population indicated that the CYP2C19*2 variant accounts for 12% of the variation in platelet aggregation after clopidogrel treatment for 7 days [106]. A recent review identified that, among patients with ischemic stroke and being treated with clopidogrel, carriers of CYP2C19 loss-of-function alleles (*2, *3, and *8) have increased risk of recurrent stroke and composite vascular events compared to noncarriers [107]. Another example can be taken from efavirenz, an antiretroviral medicine, metabolized by cytochrome P450 2B6 (CYP2B6), UDP-glucuronosyltransferase 2B7 (UGT2B7), and CYP2A6 [108]. In this study, the authors investigated SNP associations with plasma drug levels in Zimbabwean HIV-positive patients and observed elevated plasma levels of efavirenz, which may lead to toxicity in patients due to CYP2B6*6 and CYP2B6*18 mutations. In both cases, the molecular mechanism is not known. The integrated protocol suggested in Part II would be a good starting point to elucidate the mechanism of the variations in drug sensitivity and to understand the allosteric effects of the mutations.

**Figure 1:**
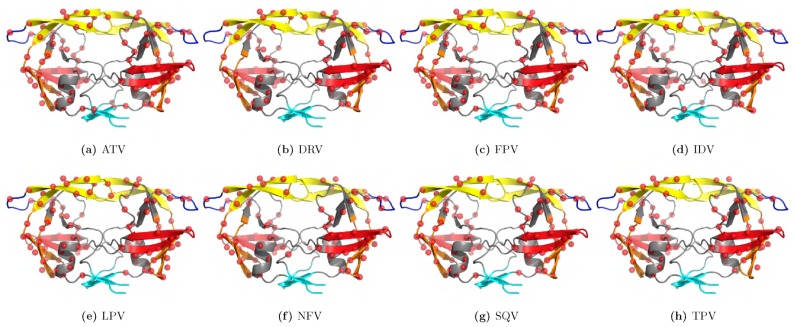
Three-dimensional mapping of variation positions for the 8 FDA-approved HIV protease inhibitors (atazanavir (ATV), darunavir (DRV), fosamprenavir (FPV), indinavir (IDV), lopinavir (LPV), nelfinavir (NFV), saquinavir (SQV), and tipranavir (TPV)) used to investigate the effects of drug resistance: Coloured cartoon representations depict the fulcrum, elbow, flap, cantilever, and interface, while the variation loci are shown as red spheres. Even though single positions are shown, some positions comprise multiple residue variations, some of which are validated drug resistance mutations (DRMs) (as per the 2017 update [114]). Figure obtained from Reference [115].

Mutations have also been associated with drug resistance in numerous pathogenic diseases such as influenza [109], tuberculosis [110], malaria [111], and HIV/AIDS [112]. A detailed understanding of pathogenicity as well as drug-resistance mechanism(s) would be essential for designing novel therapies. Sequencing the genome of pathogens can identify the drug-resistant and drug-sensitive mutations and leads to a deeper understanding of the cognate molecular mechanisms. One example can be given from a recent article by Sheik Amamuddy and colleagues [113]. The authors looked at eight Food and Drug Administration (FDA)-approved drugs and the mutations of HIV protease due to drug usage. As shown in Figure 1, the investigated mutations were interspersed within the protein (observed from collections of variants) and were not limited to the orthosteric site. While not explicitly designed to extract out the effects of specific allosteric mutations in the study, the method was able to expose underlying conserved signals buried within noisy dynamic data generated from the combined effect of orthosteric and allosteric mutations. The signals comprised a conserved and practically symmetrical lateral expansion coupled to an inward contraction captured from an ensemble of simulations. This method can easily be adapted to investigate allosteric events by designing an experiment-comprising allosteric loci, which in the case of HIV protease, for instance, could be accessory DRMs (resistance mutations not in contact with the antiretroviral drug) distal to the active site. Their approach combining MD, network analysis, and statistical calculations indicated that, regardless of the drug used, these mutations induce a common allosteric behavior in the protein in the presence of the drug, probably to dislodge the latter from the binding site. Hence, understanding the allosteric behavior of the drug targets due to mutations and identifying allosteric sites as alternative drug targeting sites would help in the design of alternative modulatory molecules. Allosteric modulators, as explained in the following section, would have many benefits over orthosteric drugs.

### 2.4. Orthosteric versus Allosteric Drugs

Orthosteric and allosteric drugs are distinguished from each other by their molecular mechanism of action. The former generally binds to the active site and is in competition with biological cofactors or substrates. Alternatively, allosteric drugs and modulators can modify or obstruct the active site when bound to an allosteric site. As allosteric binding sites are significantly less conserved compared to orthosteric sites, the principal advantage of allosteric drugs lie in their higher specificities and thus lower risks of toxic side effects [116,117,118,119]. In contrast, orthosteric site conservation allows an orthosteric drug to bind not only to the intended protein but also to unintended homologous protein family members, contributing to adverse side effects. Off-target toxicities are widespread amongst anticancer drugs in clinical trials [120]. Allosteric sites are also highly enriched in hydrophobic residues, whereas orthosteric sites are enriched with polar residues [43]. A study by Smith and colleagues hinted at the higher rigidity and aromaticity of allosteric modulators [121], thus providing an additional criterion for selecting modulators that are more likely to bind to an allosteric site. Allosteric modulators induce conformational changes and distal effects on the orthosteric site residues [122]. Understanding the structural and molecular mechanism of the induced effects is important for the rational design of allosteric modulators and site identification.

Orthosteric drugs have been successfully used in therapy, for instance, to reduce viral loads in HIV patients to undetectable levels [123]. Unfortunately, the selective pressures of drug treatment have brought forth associated transmissible drug resistance mutations (DRMs), which render these same drugs less effective over time, thus constraining future treatment strategies [124]. Some critically important examples of drug resistance concerns include the emergence of extensively drug resistant tuberculosis [125], where typical treatment options cease to work, and the recent surge of multidrug drug-resistant HIV across Africa, America, and Asia [126]. The last report issued by the Centers for Disease Control and Prevention listed 18 antibiotic resistant pathogens, including those resistant to many commonly prescribed antibiotics [127]. The discovery and use of novel allosteric drugs may therefore open the door for a wider array of future therapies as their more diverse loci could alleviate selection pressures from the currently low-performing orthosteric drugs.

Targeting allosteric sites of functional proteins in *Mycobacterium tuberculosis* (Mtb) has played a key role in research towards alleviation of TB. The protein pyruvate kinase was observed to exhibit some allosteric mechanism and deemed an attractive target of Mtb therapy [128]. Similarly, an allosteric inhibitor was identified for the Mtb enzyme ornithine acetyltransferase [129] and yet another for the enzyme tryptophan synthase [130]. Bacterial and viral infection agents can be targeted by looking at proteins common to them. Rab II is a protein from the Rab family of GTPases and has been identified as important in a number of disease-causing agents, including influenza A virus; pneumonia, caused by *Chlamydia pneumoniae*; and *Chlamydia trachomatis*, which causes a number of sexually transmitted infections by associating with other proteins to facilitate transport processes in the cell [131,132,133]. This protein has been shown by Kumar et al. to possess allosteric-binding sites, and its allosteric inhibition would be an effective remedy option towards eradication [134]. Another good example showcasing the relevance of allosteric research in disease alleviation is the case of the American trypanosomiasis (Chagas disease), where pockets with allosteric potential were identified and characterized in a cysteine protease (cruzain) in efforts to inhibit the causative agent *Trypanosoma cruzi* [135].

### 2.5. FDA-Approved Allosteric Drugs

Protein allostery research has been ongoing for years, and our understanding of protein allosteric modulation is strengthening [43]. Publicly available data concerning protein allosteric sites and their modulators is steadily increasing—the AlloSteric Database (ASD) now holds over 1900 protein targets and more than 82,000 allosteric modulators [136]. Despite continually growing investment in allosteric research, only 19 of these modulators are approved drugs compared to the current total of more than 3700 approved drugs [136,137], almost all of which bind to an orthosteric site [138]. This comparison exposes the difficulty of allosteric drug discovery. The paucity of allosteric modulators most likely results from a mixture of their reduced binding affinity, their relatively higher hydrophobicity compared to orthosteric ligands [139], and their frequently adverse structure–activity relationships [30]. Furthermore, the discovery of initial hit compounds is impeded by the difficulty in detecting allosteric binding sites as well as the shortage of knowledge of allosteric interactions and their consequences for protein modulation [43]. To date, only one approved allosteric drug has been discovered solely using in silico methods, namely enasidenib (Table 1). Overall, there is a substantial need for the elucidation of protein–modulator interactions in allosteric binding.

## 3. Part II: An Integrated In Silico Approach for Allosteric Drug Discovery

### 3.1. The Main Workflow

**Figure 2:**
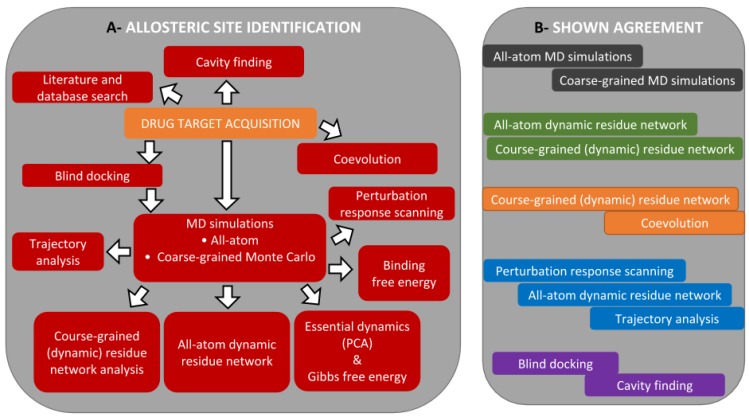
(**A**) Our proposed integrated workflow for allosteric site identification, which starts with the acquisition of a drug target and (**B**) different concepts and techniques from molecular simulation that can provide correlating information to discover and characterize allosteric events in proteins.

**Figure 3:**
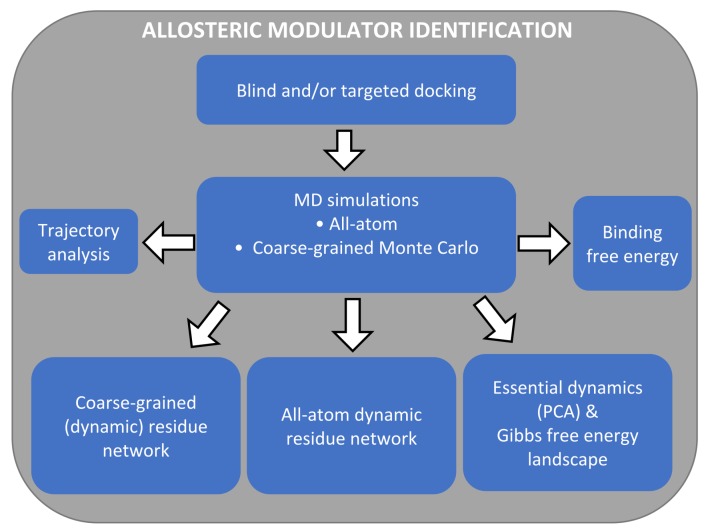
Our proposed integrated workflow for identifying allosteric modulators.

Central to this work, we propose an integration of in silico approaches that can be used for allosteric drug discovery (Figure 2 and Figure 3). The proposed workflow has been developed as a result of our research over the last few years, as indicated below and in the sections that follow. The workflow diagram gives a complete picture of all techniques, but in a particular case, only some of the approaches would actually be used. Our holistic approach starts with the acquisition of a high-quality drug target and goes through a series of approaches for allosteric site prediction before finally proceeding to the identification of allosteric modulators. We emphasize that the structural reliability of the chosen target is of utmost importance for the success of the subsequent steps. As done for most in silico allostery detection approaches, we start by finding putative allosteric binding sites before zooming onto them to determine and characterize possible binders by analyzing stabilities from conformational sampling processes. As many of the approaches and methods are common between allosteric site and allosteric modulator identification, we will follow the order given in Figure 2A while introducing them. Some of the approaches were shown to be in agreement with each other (Figure 2B). These will be indicated where necessary.

Some examples of the use of the proposed workflow to investigate allosteric phenomena in proteins of medical significance are as follows: (1) Perturbation response scanning (PRS) was combined with all-atom MD and DRN to investigate the allosteric potential of remote residues to effect conformational changes [35] and has been supplemented with docking to investigate the distal effects of ligand binding [29] in human Hsp protein; (2) homology modelling, docking, MD, essential dynamics, and DRN were jointly used to identify allosteric modulators of malarial Hsp, after which coarse-grained conformational sampling simulations coupled to residue network analysis showed corroborating results, obtained from a fraction of the computation time required compared to the all-atom simulations [33]; and (3) allosteric effects associated to alpha carbonic anhydrase SNVs were discovered by coupling all-atom MD to DRN—further characterization was achieved using essential dynamics and binding free energy landscape analysis [96]. As can be observed from these examples, one has a better chance of discovering and characterizing allosteric events by combining multiple techniques, among which simulating protein motion and coupling to DRN appear to be very effective.

### 3.2. Allosteric Site and Modulator Prediction

### 3.3. Drug Target Acquisition

As a first step for in silico allosteric modulator identification, one requires a target protein. Three-dimensional structures for the investigated target may already be available fully or in part from the PDB [159]. In the absence of an experimental structure, a computational modelling approach, such as homology modelling, can be used to model an entire protein. Further homology modelling may be useful to insert non-synonymous residue variations using experimental structures as templates, but it can also be used to impute missing ones from incomplete experimentally determined structures [86]. Over the years, the Research Unit in Bioinformatics group developed detailed homology modelling approaches, and the methodology is given in a number of group articles [32,33,35,160,161,162].

There is a long list of user-friendly web servers available for modelling protein 3D structures, some examples of which include I-TASSER [163], ModWeb [164], Phyre2 [165], PRIMO [166], RaptorX [167], Robetta [168], HHPred [169], and SWISS-MODEL [170]. MODELLER [171] can be used as a stand-alone tool by the more technically inclined users or when more customised solutions are needed, such as loop refinement or when large volumes of modelling jobs are to be calculated. As a last resort for target domains with no detectable homologs, ab initio modelling may be an option [172,173]. However, compared to the aforementioned target acquisition approaches (threading and homology modelling), the latter is accurate only for small proteins [174,175]. More recently, a command-line protein structure prediction tool based on a recurrent neural network has been developed, showing promising accuracies and producing models in a fraction of the time using only a position-specific scoring matrix and the target sequence as inputs [176]. Finally, before the structure can be used in the subsequent steps, one should validate the quality of the models via local and global metrics. ANOLEA [177], QMEAN [178], ProSA [179], Verify3D [180], and ModFOLD [181] are a few examples used to determine local quality scores, while z-DOPE [182], resolution, and QMEAN are a few examples of global metrics. Using the MODELLER tool, a per-residue profile of the z-DOPE can also be obtained for pinpointing poorly modelled regions [183]. As no single evaluation criterion is perfect, one should investigate multiple metrics to ascertain the validity of the model. As a general rule in homology modelling, (1) the template resolution should be as high as possible, while the R-free and R-value should be minimal, as they are the residuals between experimental diffraction data and the predicted crystallographic model, and (2) the environmental conditions should closely match those that are to be experimented on [184], such as the presence of a co-crystallized ligand and the receptor protonation states. Unfortunately the presence of certain molecular entities may require some prior planning due to the incomplete transferability across certain force fields [185] for use in downstream approaches (such as MD). Finally, given that differences in receptor conformation specify different topologies and alter the ligand-accessible surfaces, it is clear that this plays a major role in experimental designs aimed at discovering allosteric sites.

#### 3.3.1. Mining Literature and Databases for Allostery Information

Before attempting to identify an allosteric site of a protein, it is good practice to determine whether the literature already describes it from previous work. The AlloSteric Database (ASD), which hosts a collection of known allosteric targets and their modulators, is a major source of relevant literature and annotations pertaining to the concept of allostery [136]. The database has grown to become a central resource for the storage, retrieval, and analysis of allosteric protein datasets [136,186,187]. It organizes information about allosteric regulation by receptor target and modulator (activator, inhibitor, or regulator), and entries incorporate additional annotations such as the associated interactions, sites, pathways, functions, and any linked disorders [136]. A good example of the use of the ASD in allostery research can be drawn from work done by Astl and Verkhivker [188]. Using information retrieved from the database, they combined coarse-grained MD and residue interaction network to demonstrate the differential effects of allosteric inhibitors and activators on the global dynamics and network organizations within protein systems, comprising 300 diverse proteins and complexes. In the case of insufficient information characterizing allostery within the chosen target protein, one can proceed to cavity-finding approaches for the discovery and characterization of *de novo* allosteric sites.

#### 3.3.2. Cavity-Finding Approaches

Pockets/cavities present in proteins might be functionally important and have been recognized as conventional sites for ligand binding [189]. For those targets that are not well characterized for such pockets, the identification of these sites is thus a starting point for potential allosteric-site prediction and structure-based drug design, with the effects of ligand binding confirming allostery [190]. There is a wide array of freely available cavity-finding tools, mostly in the form of web servers which use a variety of approaches to provide potential starting points for the discovery allosteric sites. The methods employed include, amongst others, the use of NMA, energy evaluations, machine learning, and MD simulations. As features characteristic to allostery are not completely understood, no single predictive method can be exhaustive and it is best to utilize a combination of approaches to increase the level of support for the potential sites. Some of the most recent cavity-finding approaches are summarized in Table 2.

#### 3.3.3. Blind Docking

Small-molecule docking to a target protein can give us an indication as to whether and how strongly a ligand might bind to a certain surface, but limiting the search space to a primary binding site (i.e., targeted docking) can be oblivious to allosteric pockets. In blind docking (BD), the whole protein surface is scanned for putative binding sites and is especially useful when one has not been determined a priori [191,192,193]. Several studies have used BD for allosteric site identification, for which we give some examples in this section. Iorga and colleagues have used BD of three known allosteric modulators to reveal putative binding sites from the acetylcholine-binding protein and in homology-modelled human nicotinic receptors [194]. Grant and coworkers identified potential allosteric pockets on the Ras protein catalytic domain via BD of 267 putative ligands from PubChem BioAssay database [195]. Pavlovicz and colleagues used BD to identify negative allosteric sites on the numan α4β2 and α3β4 neuronal nicotinic acetylcholine receptors [196]. Jin and colleagues used BD with tyrosine phosphatase 1B to reveal a hydrophobic and less conserved allosteric site in contrast to its negatively charged but highly conserved active site [197]. Based on this study, an alternative drug-design strategy was proposed. Lastly, a study by Chen and colleagues employed BD to explore a specific allosteric site for non-peptidic inhibitors located behind the catalytic triad of the dengue virus-2 NS2B-NS3 protease [198].

Due to the increased search space, stochastic strategies are typically used to make the exploration of the larger target surface computationally tractable [199]. One way to overcome this limitation is to divide the search surface [200] and/or to increase the search exhaustiveness.

In their pioneering paper introducing BD using AutoDock, Hetényi and van der Spoel recommended over 100 independent docking runs with flexible ligands [192], which would be analogous to the exhaustiveness parameter in AutoDock Vina, for example. Further, in a comprehensive study evaluating ten common docking programs [201,202,203,204,205,206,207,208,209,210], Wang and colleagues showed that these tools correctly predicted the ligand poses even though the binding energies could not be estimated accurately [211]. As BD involves a larger volume to scan, the docking process can be limited by efficiencies of the sampling algorithms and complexity of the scoring functions [193]. A more accurate estimation of the ligand-binding energy was achieved via quantum mechanical (QM) and semi-empirical QM-based rescoring schemes [212,213]. This rescoring scheme was successfully applied to HIV-1 protease (with 22 ligands) [214], cyclin-dependent kinase 2 (with 31 ligands) [215], and casein kinase 2 (with 23 halogenated ligands) [216].

Key regions important for the stability of subtilisin (an industrially important serine protease) were determined using PRS, in which the covariance matrix was obtained from equilibrated portions of MD trajectories [217]. PRS was, for the first time, applied to large, flexible proteins by Penkler et al. (2017), determining key residues involved in allosteric control in the 70 kDa heat shock protein (Hsp70) [218] using an implementation of the PRS algorithm available from the MD-TASK package [97]. More recently, the same approach was coupled with DRN to determine potential effector residue loci promoting allosterically driven conformation interconversions in human Hsp90 [35] before targeting the same sites (at the C-terminus) by high-throughput virtual screening for druggable pockets [29]. After Penkler’s work showcased the application of PRS to highly dynamic proteins, Amusengeri and Tastan Bishop employed a similar workflow in their work in Hsp72 and Hsc70 to search for allosteric modulators [32] from the South African natural compounds database (SANCDB) [219]. While linearly impacting a protein allows us to promptly assess the likelihood of transitioning from a static starting state to a target state, measuring and summarising topological changes from conformational sampling processes opens up a new avenue for the detection and characterization of allosteric effects, which we describe in the next section focussed on the application network analysis in assessing protein dynamics.

#### 3.3.4. Perturbation Response Scanning

By factoring out the bound small molecule, to instead perturb single residues from a receptor and to record the linear response, is a rapid way of approximating biological perturbations in proteins. These perturbation events can be instantiated by various intrinsic and extrinsic factors, which may in turn have far-reaching effects [27,36]. In perturbation response scanning (PRS), a series of uniformly distributed forces are sequentially applied to each residue of an equilibrated protein conformation to estimate the agreement with a desired target state [226]. The perturbation is obtained from the dot product between a covariance matrix and a force vector. The covariance matrix can be obtained from various models of interatomic potential such as those used in MD [29] or by inverting a Hessian matrix [227] as typically done in ENM [226]. The uniform force vector impacts a given residue multiple times, and a correlation consensus is obtained for that residue’s overall effect against a targeted conformation. The algorithm is sequentially applied to each residue of the protein to yield a correlation value in each case. The PRS algorithm has been successfully used to uncover mechanisms of allostery in various experiments. For instance, Gerek and Ozkan used PRS to compare allosteric transitions in two PDZ domain proteins (proteins linked to cancer and to Alzheimer’s and Parkinson’s diseases) by perturbing inverted Hessian matrices obtained from ENM models [228].

#### 3.3.5. Interaction Networks in Proteins Dynamics

##### The Usefulness of Network Theory in Investigating Protein Dynamics and Allostery

As reviewed recently by Liang and coworkers, the last decade has produced an array of various tools that bridge multiple interdisciplinary concepts for the study of protein dynamics and allostery regulatory mechanisms [36]. More specifically, in allosteric signalling, the perception and long-distance relay of the trigger signal is associated with the rewiring of an intricately connected network of non-covalently interacting protein residues [36]. This ultimately leads to directly observable conformational changes and/or to entropic changes, where no conformational change is seen. Simplifying the protein topology as a network of nodes connected by edges to represent the residues and their interaction (or strength of interaction), respectively [23,229,230], allows for the investigation at various levels of the allosteric effects, which are otherwise convoluted with protein entropy. The increasingly popular integration of network theory for protein dynamic analysis thus plays a major role in robustly deconvoluting complex relational behaviours into a more interpretable and quantifiable form [230,231,232,233,234,235,236,237,238,239,240]. Several studies have pointed out the tremendous usefulness of modelling and analyzing proteins as dynamic entities oscillating between various allosteric states in order to extract meaning from the complexity of allosteric regulation [29,80,81,94,224,241,242,243,244,245].

##### Dynamic Residue Network Analysis

Networks are typically represented by either an adjacency matrix or an edge list [36]. In static networks, a binary contact is inferred between each protein residue pair (defined by C-alpha or C-beta atoms) using a defined cut-off distance (i.e., 6.7Å). The DRN approach extends this idea by computing the time-averaged version of static contact networks using MD simulation data [97]. Different variations of time-averaged networks have been used to investigate allosteric effects in various proteins, for instance, in characterizing (1) the dynamics of catalysis in Cyclophilin A variants [95], (2) in characterizing the effects of damaging non-synonymous SNVs of the renin–angiotensinogen complex [86] and carbonic anhydrase 2 [96], and (3) in investigating cross-domain allostery in human heat shock proteins [29,32]. The MD-TASK package was the first downloadable tool packaging a set of nonconventional methods meant to analyze MD simulations. These comprise a set of freely available Python scripts aimed at computing DRN metrics in addition to other scripts used for carrying out MD analysis [97]. More specifically, these network metrics comprise the time-averaged versions of both betweenness centrality (*BC*) and averaged shortest path (*L*), calculated for each protein residue (node) using MD trajectory data. While *BC* measures the number of geodesics (shortest paths between two nodes) going through an intervening node [246], *L* averages the number of geodesics inbound to a node. Both metrics gain robustness in predicting medium-to-long-distance relational information from averaged information across conformational ensembles. By computing the average geodesics between every residue pair, *L* would effectively be maximized when a structure is compact and minimized otherwise. As *BC* is evaluated for intervening residues linking pairs of residues, it gives a good indication of the importance of that residue for information flow within the protein. Penkler et al. for the first time showed that there is correlation between *BC* and 1/RMSF (root mean square fluctuation) as well as between *BC* and 1/*L* [35]. Interestingly, the article also highlighted a high correlation between PRS hot spot residues and residues with high *BC* values. This correlation, later, was also identified in other studies [32]. More recently, Kimuda coupled DRN to the molecular mechanics Poisson–Boltzmann surface area (MM-PBSA) analysis and principal components analysis (PCA) to identify 18 novel potential inhibitors of pteridine reductase 1 in *Trypanosoma brucei*—the causative agent of Human African Trypanosomiasis—five of which inhibited the pathogen’s growth in vitro [247].

In a new approach developed by Sheik Amamuddy and coworkers, a statistically guided network was able to show two coupled conformational changes associated with antiretroviral (ARV) drug resistance, using one-tailed t-tests on collections of residue pairwise distances determined from ensembles of protease structures sampled by MD [113]. Despite the presence of a multitude of nsSNVs and selected DRMs, situated at multiple loci within the viral drug target, they were able to reproducibly detect these motions in HIV proteases with a high degree of conservation across 8 FDA-approved inhibitors despite the chaotic nature of protein dynamics. This technique, which combines t-tests and the degree centrality, may hold a lot of promise for the analysis of alternate phenotypes (such as in the study of drug resistance) and may be invaluable in the characterization of allosteric effects. The recently published statistically guided network construction technique proved highly sensitive in detecting distinct motions from ensembles comprising hundreds of drug-resistant and drug-sensitive HIV protease variants, whereby present nsSNVs were dispersed within the protein structures [113]. The calculation of the degree centrality in this approach is very attractive for making inferences from batches of comparable protein variants, with potential applications in comparing allosteric effects between alternate pairs of a given phenotype.

##### Coevolution and Residue Interaction Networks

A functional site that mediates communication pathways and determines organization of the residue interaction networks often coincides or tightly couples with coevolving residues. Statistical coupling analysis (SCA), mutual information (MI) model, and covariance-based approaches have employed sequence-based analysis of residue coevolution in homologous families to show that functional residues in residue networks are connected via strong evolutionary relationships [248,249,250,251,252,253,254,255] Coevolution of protein residues can reflect correlated functional dynamics of these sites in mediating residue–residue contacts [256], protein folding transitions [257], and allosteric signalling in protein complexes [258]. Coevolving residues could also form direct communication paths in the interaction networks with connections weighted according to dynamic couplings and coevolutionary interaction strengths between nodes [259,260,261]. Structurally stable and quasi-independent modules of physically interacting coevolving residues (protein sectors) appeared to play a key role in mediating protein stability and long-range interactions [250,251]. Several computational methods have been developed to evaluate the extent of mutual information and coevolutionary dependencies between residue pairs [252,253,262,263,264]. Computational analysis of residue interaction networks and community analysis have shown that local dynamic modules anchored around functional residues can serve as building blocks to connect distant functional regions and to mediate allosteric conformational transitions [94,252,265,266,267,268]. Dynamic and coevolutionary residue correlations may also act as synchronizing forces that determine modular organization of allosteric interaction networks and enable efficient allosteric regulation [94,269,270]. These results have motivated the development of novel community-based methods for modelling ensembles of allosteric communication pathways in protein structures [94,269,270]. Using this computational framework, it was found that efficient allosteric communications in various signalling proteins could be controlled by structurally stable functional centers that exploit dynamically coupled residues in their local communities to propagate cooperative structural changes.

After having scanned the structure of interest using the allosteric site prediction approaches to obtain various sources of support for putative ligand binding sites, a consensus can be drawn before proceeding to the identification of allosteric modulators.

#### 3.3.6. Conformational Sampling

##### Molecular Dynamics

MD is an invaluable tool in the hands of protein allostery researchers [44]. MD experiments simulate biological protein movement at various levels of theory, for which molecular mechanical approximations (coupled to Newton’s second law of motion) [271] are the most common for relatively large solvated protein systems. At the junction between the quantum and coarse-grained atomic models, all-atom MD simulations provide a good trade-off between accuracy and speed for conformational sampling, producing quality spatiotemporal data associated to protein action. To investigate allostery, a researcher would typically dock a prospective binder against a protein of interest before characterizing any ligand or protein changes. For such work, conventional methods of analysis can be supplemented by DRN (Part II, Section 3.3.5). These methods can also be used to study the allosteric effects of protein variants. Utilizing sequence data, it is possible to elucidate the mechanisms associated with disease-causing sequence variants using protein dynamics and allosteric regulations [36,272]. All-atom MD may not be able to sample enough conformations under reasonable computation time in the case of rare events or for changes that occur over longer periods. Coarse-graining simulations and accelerated sampling approaches are the next best approaches and are discussed in the next section.

##### Coarse-Grained Simulations and Stochastic Markov State Models

Coarse-grained models are computationally effective and enable simulations of long timescales for large allosteric systems and assemblies, thereby allowing for observation of allosteric structural and dynamic changes [33]. Functional and large-scale flexibility changes in allosteric systems can be predicted using several popular coarse-grained methods such as CABS-flex [273,274,275,276], NMSim [277], and FlexServ [278]. CABS-flex uses a coarse-grained model in combination with an efficient search protocol [274,275] and allows the generation of trajectories that can accurately recapitulate the all-atom MD simulations for long-time processes [274,275,279]. NMSim is a computationally efficient alternative to all-atom MD simulations and can be employed for sampling of large conformational space and pathway generation [277]. FlexServ method and server provides access to three coarse-grained algorithms for simulations of protein flexibility: discrete dynamics, NMA, and Brownian Dynamics [278]. These coarse-grained approaches provide robust and efficient means for simulation and analysis of large conformational changes and allosteric transitions in protein systems that otherwise are difficult to observe in all-atom MD simulations. ENM are a type of coarse-grained NMA, which can substantially reduce the computational demands to efficiently explore protein dynamics around a single energy minimum by simplifying interatomic interactions as spring-connected nodes (protein C${}_{\beta }$ and/or C${}_{\alpha }$ atoms) [227,280,281,282,283,284,284]. Several studies involving this approach have verified its success in identifying functionally relevant protein conformations [175,285,286,287,288,289,290,291,292]. Further, coarse-grained NMA [293,294,295] and ENM integrated with the information-based Markovian theory of signal propagation [296,297,298,299,300,301,302,303] have provided a generalized formalism of allosteric communication pathways in proteins.

Given the complexity of thermodynamic and kinetic factors underlying allosteric regulatory events, stochastic MSMs [304,305,306,307,308,309,310] have become increasingly useful states-and-rates network models with the software infrastructure [311,312,313,314] for describing the probability of transitions between functional states during allosteric events [77,315,316]. Combined with MD simulations, MSM approaches can provide detailed network connectivity maps of states on the free-energy landscape and can estimate the effect of allosteric perturbations on the conformational equilibrium and kinetics of allosteric transitions. Rare transition events between long-lived states are a key feature of allosteric proteins that are difficult to observe in direct MD simulations because of the long simulation timescales needed. To circumvent these limitations, MSMs can be built from multiple shorter simulations yet describe long timescale dynamics accurately. While protein structure network models describe allosteric interactions between residue nodes, MSM network maps are markedly different by representing discrete protein states as the system nodes. The connections between Markovian state modeling and network-centric analysis of protein structure have never been rigorously explored for understanding of allosteric processes, leaving a significant conceptual void in the current knowledge and the existing repertoire of computational approaches. The integration of the experiment-informed Markovian modeling of protein dynamics and the information-theoretical description of dynamic flows and entropy transfer in the networks of protein states represents an interesting and promising avenue for further exploration of allostery. Flow-based model methods [317,318,319,320,321,322,323,324,325] operate through a stochastic walk on the dynamics of the network rather than on its topological structure, where communities consist of dynamically interconverting conformations among which the dynamic flow can persist for a long time and define functionally significant states. The apparatus of the map equation can reveal modular patterns in the dynamic flows of allosteric proteins, allowing to map conformational transformations between allosteric states and to reconstruct regulatory mechanisms.

In the following section, we describe some analysis strategies that have shown their efficacy in investigation of allosteric effects using data obtained from conformational sampling.

#### 3.3.7. Trajectory Analysis

After performing preliminary quality checks on the simulated protein dynamics data, conventional metrics such as the root mean square deviation (RMSD), residue root mean square fluctuation (RMSF), radius of gyration (Rg), and geometry calculations (distances and angles) can be performed to infer the stabilities (or instabilities) within protein–ligand complexes for the previously docked compounds [29,32,233,326,327]. Distal effects of interfacial residue variations within the renin–angiotensinogen complex were uncovered by Brown and colleagues using RMSF alongside *BC* [86]. Dynamic cross correlation (DCC) is an additional option for characterizing the residue motions sampled from MD simulations, an example of which can be found from work done by Bowerman and Wereszczynski to characterize the allosteric effects of a thrombin antagonist [328]. An implementation of a DCC algorithm is available from MD-TASK. For a bird’s eye view of the distribution and clustering of sampled conformations, essential dynamics (ED) can be supplemented with energies in free-energy landscape (FEL) calculations to analyze the stability of protein complexes under various conditions [329,330]. More specifically, it has been used by Amusengeri and Tastan Bishop to investigate the effects of allosteric ligand binding in human heat shock proteins [32].

As each of the aforementioned methods highlights different facets of simulated protein dynamics, we provide some details about their principles and the types of information that can be retrieved using such techniques. In each case, the proteins or complexes are assumed to be free from periodic boundaries and corrected from rotational and translational effects. In the following section, we describe several approaches that can be used for analyzing results obtained from simulated protein dynamics.

##### RMSD

The root mean square deviation evaluated between a target and a given reference structure gives an idea of the overall deviation of all atoms of the structure from those of the reference. The RMSD evaluated at time $t_{i}$ is calculated as shown below, where *N* is the number of atoms, *m* is the atomic mass, *x* is the generalized coordinate, and $t_{ref}$ is the frame used as reference, being generally the frame at t=0:

(1) $$RMSD\left(t_{i}\right)=\sqrt{\frac{1}{N}\sum_{j=1}^{N}m_{j}{\left[x_{j}\left(t_{i}\right)-x_{j}\left(t_{ref}\right)\right]}^{2}}$$

For an MD simulation, it is important that the structures are properly aligned such that correct distances are evaluated. As it is often the case that trajectory-specific topologies are used in independent simulations, care should be applied when interpreting and comparing pairs of such systems, for instance, in an allosteric modulator-bound and a modulator-free protein or in a wild-type and a variant, as each protein may have a slightly different starting conformation as reference. Work by Penkler and Tastan Bishop comparing allosteric effects from ligand-bound and ligand-free Hsp90α clearly suggests the presence of multiple receptor conformations, as seen from the multimodal distribution of RMSD data [29].

##### RMSF

While RMSD calculates an average obtained from a collection of atom coordinates, an RMSF value is computed for each atom as the standard deviation of atomic fluctuations recorded over time. For each atom, the RMSF gives an estimate of its variability around its time-averaged value, once more from aligned conformations. Comparing RMSF values for homologous residue positions across two systems thus allows for the local inspection of differential residue flexibility, given an allosteric perturbation.

(2) $$RMSF\left(x_{i}\right)=\sqrt{\frac{1}{T}\sum_{j=1}^{T}\left[x_{i}\left(t_{j}\right)-{\bar{x}}_{i}\right){]}^{2}}$$

As an example of the use of RMSF for the detection and partial characterization of allosteric effects, Brown and coworkers observed increased rigidities across several deleterious variants of the renin-angiotensinogen complex with respect to the wild-type complex at sites distal to the dimer interface where known nsSNVs had been introduced [86].

##### Radius of Gyration

In the calculation of Rg, radii are first computed between each atomic ($r_{j}$) center of mass (COM) and the molecular COM ($R_{i}$), before computing the RMSD to give an indication of the degree of compactness for a protein at a given frame *i*.

(3) $$Rg\left(t_{i}\right)=\sqrt{\frac{1}{N}\sum_{j=1}^{N}m_{j}{\left[r_{j}\left(t_{i}\right)-R\left(t_{i}\right)\right]}^{2}}$$

A higher averaged radius hints at a less compact structure. For instance, a residue mutation situated at the interface between two interacting protein surfaces may destabilize them and increase the Rg value. Unless the protein conformational changes are large enough, one may not always find significantly different Rg distributions. In such cases, one may focus on more localized regions, such as an individual domain for example. Sheik Amamuddy and coworkers used Rg distributions as one of their methods to investigate drug-resistance-related changes from ensembles of drug-resistant and drug-susceptible HIV protease structures of the B subtype [113].

##### Dynamic Cross Correlation

In essence, DCC performs pairwise correlations of atomic motion, generally by normalizing the covariance matrix over the product of the respective standard deviations of atomic fluctuations recorded over time for each atom pair. Provided the simulations are not too short, this allows one to compare motions that trend apart or together within a single system. As for the other metrics, it can be used to compare analogous systems that have experienced a given allosteric perturbation to determine correlative properties from the experiment.

##### Geometry Calculations

Geometry calculations comprise the calculations of distances, bond angles, and dihedrals. For example, one may compare the domain–domain distance in a multi-domain protein or the inter-residue distances resulting from the introduction of a non-synonymous residue mutation or the binding of a ligand at a site away from the active site. In an experiment investigating allostery-related effects in the redox-dependent conformational dynamics in the multidomain human protein disulphide isomerase (hDPI), Karamzadeh and coworkers computed several geometric features, such as the inter-domain angles and distances, torsion angles, and pairwise residue distances in order to build machine-learning classifiers able to discriminate between the redox states [331].

##### Essential Dynamics and Free-Energy Landscape

The application of ED to analyze protein behaviour mainly focuses on the dominant modes of motion, which are obtained by decomposing the covariance matrix and by ranking the eigenvectors (principal components) in descending order of the corresponding eigenvalues [292,332]. By plotting the first two or three principal components against each other, we obtain a general idea of the distribution for all generated conformations obtained from a conformational sampling experiment, as exemplified from ED analyzes for various proteins [333,334,335,336]. On the resulting figure, one may obtain conformationally distinct clusters of the simulated proteins or complexes. When supplemented with energy levels, the 3D FEL plot effectively highlights areas of low and high energies, which typically correspond to the metastable and transitory states respectively. According to the principle of minimal frustration, troughs in the landscape are generally populated by the more native conformations [337]. Given an allosteric signal, the population of protein conformations can be shifted to an alternate state against an otherwise higher energy barrier. The FEL is thus a very important tool in investigating the allosteric effect, when conformational changes are present, as exemplified by a case study showcasing the application of computational methods to discover allosteric modulators in malarial proteins [33], and in the characterization of Discorhabdin N as potential allosteric modulators of anticancer drug targets [32].

## 4. Conclusions

While we have gained important insights into the function of allosteric proteins, the quantitative characterization of these highly dynamic and often elusive processes continues to present formidable technical and conceptual challenges. Allosteric events in biological systems occur on different spatial and temporal scales and involve a complex interplay of thermodynamic and dynamic changes that are difficult to observe, simulate, and interpret.

Experimental studies of protein systems indicate that allosteric regulation may involve a combination of the classical models of allostery, i.e., conformational selection, dynamic allostery, and induced fit. An understanding, at the structural level, of the relationships between protein robustness, allosteric drug binding, and disease may be of use in the development of theoretical and experimental approaches bridging structure-based network analysis of protein targets with modelling of protein interaction networks and pathways. The complexity and diversity of these processes require innovative theoretical and data-driven approaches that can bridge advances in structural and quantitative biology in transformative yet practical ways. The development of novel integrated research strategies should address these challenges by strengthening and advancing the interface between molecular biophysics, network biology, and data science. We argue that the next breakthrough in the discovery of allosteric drugs may require such integration of traditional biophysical approaches with systems biology and experiment-guided machine-learning tools to bridge a detailed microscopic analysis with macroscopic modelling of allosteric phenomena in cellular networks and signalling pathways.

## Figures and Tables

**Table 1:** List of currently approved allosteric drugs [136] in alphabetical order.

Drug/Code Name	Medical Condition	Mechanism	Enzyme Target	Discovery Method	2D Structure
Carglumic Acid	Acute hyper- ammonaemia	Activator	Carbamoyl phosphate synthetase 1	Experiments in rats, both in vivo and in vitro [140]	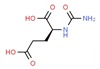
Cinacalcet	Hyper- parathyroidism	Activator	G protein- coupled receptor	Functional responses of cells regulated by calcium receptor activity: PTH secretion by parathyroid cells, calcitonin secretion by C-cells, and bone resorption by osteoclasts. [141]	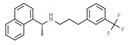
Clonazepam	Epilepsy	Activator	γ-amino- butyric acid (GABA)	Perifused frog neuro- intermediate lobes [142]	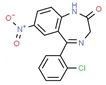
Cobimetinib	Melanoma	Inhibitor	MAPK1, MEK1 & MEK2	Structural insight—manipulation of previously known MEK inhibitors’ structure. Ligand- binding affinity assays [143]	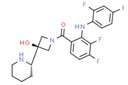
Cyclothiazide	Hypertension	Activator	AMPA Receptor	AMPA- and KA-induced [3H]NE release from slices of rat hippocampus [144]	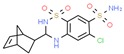
Drotaverine	Irritable bowel syndrome	Inhibitor	L-type Ca^2+^ channel	Saturation studies. Dissociation kinetics [145]	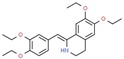
Enasidenib	Acute myeloid leukemia	Inhibitor	IDH2	In silico: Binding free energy, conformational change [146]	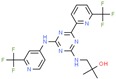
Flurazepam	Insomnia	Activator	GABA-A receptor	Site-directed mutagenesis. Concentration-response analysis [147]	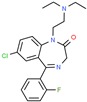
Ivermectin	Parasite infestations	Activator	Alpha7 neuronal nicotinic acetylcholine receptor	Mutagenesis. Cell line, culture, and recordings [148]	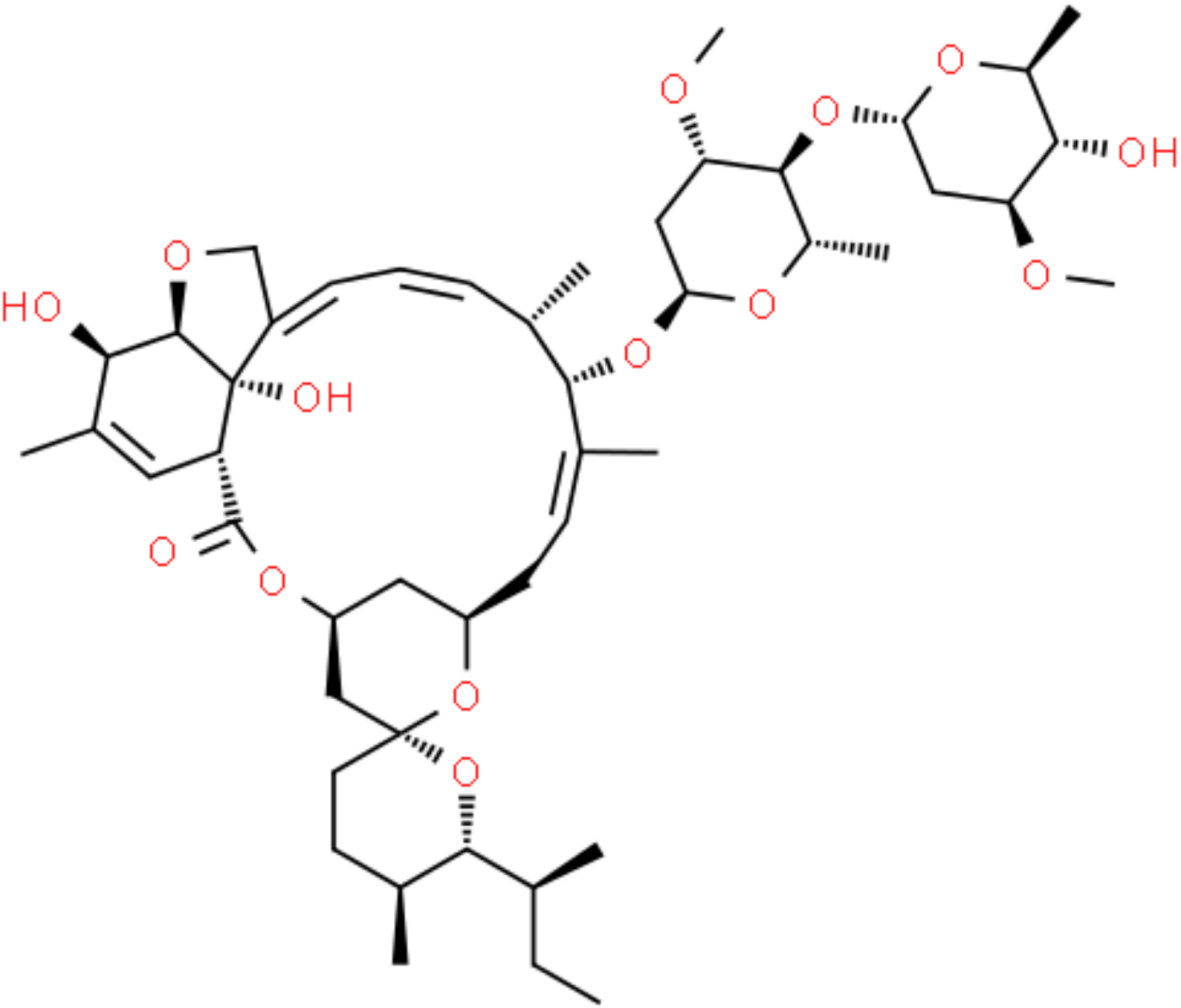
Ketazolam	Anxiety disorder	Activator	GABA-A receptor	Increase of GABA level in cat spinal cord and in the total brain of mice and rats [149]	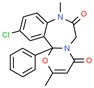
Lorazepam	Anxiety disorder	Activator	$\alpha_{1}$-adrenergic receptor	Transfection. Ligand-binding affinity assays [150]	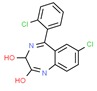
Maraviroc	HIV	Inhibitor	C-C chemokine receptor type 5	Displacement binding assays. Dissociation kinetics [151]	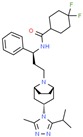
Niclosamide	Neuropathic pain	Inhibitor	Group 1 metabotropic glutamate receptor	Calcium mobilization assays. Cross-receptor selectivity experiments. Computati- onal molecular modeling analysis. NP-evoked mechanical hyperalgesia model in rats [152]	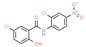
Piracetam	Dementia, vertigo, cortical myoclonus, dyslexia, and sickle cell anemia	Activator	AMPA Receptor	Enzyme crystallization. Crystal structure determination. Structure analysis [153]	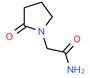
Rifapentine	Tuberculosis	Inhibitor	DNA- dependent RNA polymerase	Site-directed mutagenesis. In vitro transcription. RFP binding assays [154]	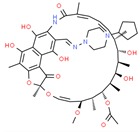
Rilpivirine	HIV	Inhibitor	HIV-1 reverse transcriptase	X-ray crystallo- graphy. Molecular modeling. Optimizing lead compounds [155]	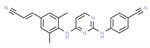
Sirolimus	Immuno- suppressive	Inhibitor	FK Binding Protein-12	Site-directed mutagenesis. FKBP12- Rapamycin (Sirolimus) binding assays [156]	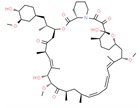
Ticagrelor	Stroke; Acute coronary syndrome undergoing percutaneous coronary intervention	Inhibitor	G protein- coupled receptor	ATP analogue production. Platelet inhibition and patient outcome (PLATO) trial [157]	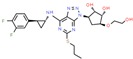
Trametinib	Melanoma	Inhibitor	MEK1 & MEK2	Enzymatic and cellular studies. Pharmacokinetic analysis [158]	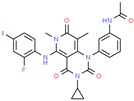

**Table 2:** Web servers for the prediction of allosteric sites.

Web Server and URL	Functionality	Input	Output
AlloDriver [220]	Identifies potential driver mutations implicated in cancer and maps them to binding sites.	A list of annotated cancer-related mutations.	Returns a list of ranked driver mutations annotated by residue loci, scores and binding site (allosteric and orthosteric), amongst many other features.
AlloFinder [221]	AlloFinder identifies possible allosteric sites via dynamic perturbations and algorithms present in Allosite. It also screens for possible binders against the identified sites. Protein-ligand complexes are then scored using Alloscore algorithms.	The receptor PDB file and a ligand library.	Displays protein-ligand complex for docked ligands within the putative allosteric site. Further, a table reports the volume of the predicted allosteric site, the perturbation score, the drug-like score, the allosteric site score and the AlloScore score. Additionally, the top 100 potential allosteric ligands are ranked according to their Alloscore. Finally, the predicted site and the predicted ligands are mapped using allosterome data.
AlloPred [82]	Uses NMA to identify potential allosteric pockets.	The receptor PDB file and active site residues.	Displays protein structure and a list of pockets with Allopred and Fpocket rankings as well as NMA effect per residue.
Alloscore [222]	Uses a linear combination of non-bonded interaction terms, a deformation term and geometric features to predict the binding affinities of protein-ligand interactions.	The receptor PDB file and a pre-docked ligand MOL2 file.	File with potential ligands and their allosteric interactions (hydrogen bonds, van der Waals, hydrophobic interactions and Alloscore values).
AlloSigMA [223]	Calculates energetics of allosteric signalling resulting from ligand binding, mutations or a combination of the two.	The receptor PDB file.	The allosteric free energy profile, colouring residues according to difference in free energy between the ligand bound and the apo-protein.
Allosite 2.0 [85]	Predicts allosteric sites by means of pocket-based analysis and support vector machine (SVM) classifier algorithms.	The receptor PDB file.	Window showing the structure and identified potential allosteric sites. Pockets can be viewed on the displayed protein structure. Properties of the pocket include: (i) Its volume, (ii) Total solvent-accessible surface area (SASA), (iii) Polar SASA and (iv) Druggability score
AllosMod [84]	Makes use of MD simulations and energy landscapes to identify allosteric conformational changes.	The receptor PDB file and its sequence.	Returns a zipped file of further input files to be MD-run by the user via MODELLER and analysed using a provided Python script.
Cavity (Submodule of CavityPlus) [190]	Identifies cavities and provides their respective drug scores.	The receptor PDB file.	Displays the structure, potential cavities and constituting residues with their respective drug scores, which determine cavity druggability.
CorrSite (Submodule of CavityPlus) [190]	Identifies possible allosteric sites from those picked up by CavityPlus on the basis of correlated motion between allosteric and orthosteric cavities.	PDB file of a proposed orthosteric site or predetermined cavities obtained from the Cavity tool.	Displays the structure with mapped orthosteric and allosteric sites. Cavities are labelled with their corresponding correlation scores to the orthosteric site.
CovCys (Submodule of CavityPlus) [190]	Identifies druggable cysteine residues for covalent allosteric ligand design.	Cavities identified by the Cavity web server.	Maps any of the selected sites onto the protein structure and displays a table of Cys residues labelled by cavity ID, targetability, pKa value, exposure and their pocket binding affinity.
DynOmics ENM [83]	Predicts allosteric communication using ENM.	The receptor PDB file.	(i) JSmol window showing structure color-coded by the size of motions driven by the slowest two modes, lowest mobility (blue) to highest mobility (red) regions, (ii) Molecular motions animation, (iii) Mapped RMSF, (iv) 3D and 2D display of selected modes, (v) Cross correlations between residue fluctuations, and (vi) Inter-residue contact maps
MCPath [224]	Identifies regions in a protein structure which may function in allosteric communication using a Monte Carlo-based approach.	The receptor PDB file and pathway data (initial residue index, length and number of paths).	List of all pathways ranked according to their probabilities and populated pathways. 3D structure onto which the top three populated pathways and their residues are mapped.
PARS [81]	Uses NMA to identify possible allosteric pockets which, upon binding of a ligand, cause a regulatory effect in the protein.	The receptor PDB file and its sequence.	Table with identified pockets ranked according to their potential as allosteric sites.
SPACER [80]	Combines ENM and docking to predict allosteric communication.	The receptor PDB file.	List of ligand binding sites, for which the following can be explored: (i) Local closeness - the output structure is colored according to surface local closeness values, (ii) Binding leverage - quantifies the cost of the binding site deformation in the presence of a ligand, and (iii) Characteristics of the communication strength between a putative allosteric site and another binding site.
STRESS [225]	Identifies allosteric hotspot residues which result in large protein conformational changes when bound by a small ligand.	The receptor PDB file.	Ranked list of predicted sites each with an index of the binding site obtained from Monte Carlo simulations, a binding leverage score and their respective residues.
